# α-Synuclein Overexpression Increases Dopamine D2/3 Receptor Binding and Immune Activation in a Model of Early Parkinson’s Disease

**DOI:** 10.3390/biomedicines9121876

**Published:** 2021-12-10

**Authors:** Kathrine Stokholm, Majken Borup Thomsen, Jenny-Ann Phan, Line K. Møller, Cecilie Bay-Richter, Søren H. Christiansen, David P. D. Woldbye, Marina Romero-Ramos, Anne M. Landau

**Affiliations:** 1 Department of Nuclear Medicine & PET-Center, Institute of Clinical Medicine, Aarhus University, 8000 Aarhus, Denmark; kathrine_st@hotmail.com (K.S.); majken.thomsen@clin.au.dk (M.B.T.); jenpha@rm.dk (J.-A.P.); linekm@gmail.com (L.K.M.); 2 NEURODIN, CNS Disease Modeling Group, Department of Biomedicine, Aarhus University, 8000 Aarhus, Denmark; mrr@biomed.au.dk; 3 Translational Neuropsychiatry Unit, Institute of Clinical Medicine, Aarhus University, 8000 Aarhus, Denmark; cbr@clin.au.dk; 4 Department of Neuroscience, University of Copenhagen, 2200 N Copenhagen, Denmark; schri@sund.ku.dk (S.H.C.); woldbye@sund.ku.dk (D.P.D.W.)

**Keywords:** adeno-associated viral vectors, α-synuclein, autoradiography, dopamine, neuroinflammation, synaptic vesicle glycoprotein 2A, Parkinson’s disease

## Abstract

Progressive degeneration of dopaminergic neurons, immune activation, and α-synuclein pathology characterize Parkinson’s disease (PD). We previously reported that unilateral intranigral injection of recombinant adeno-associated viral (rAAV) vectors encoding wild-type human α-synuclein produced a rat model of early PD with dopamine terminal dysfunction. Here we tested the hypothesis that decreases in dopamine result in increased postsynaptic dopamine D2/D3 receptor expression, neuroinflammation, and reduced synaptic vesicle glycoprotein 2A (SV2A) density. Rats were injected with rAAV encoding α-synuclein or green fluorescent protein and subjected to non-pharmacological motor tests, before euthanization at 12 weeks post-injection. We performed: (1) in situ hybridization of nigral tyrosine hydroxylase mRNA, (2) HPLC of striatal dopamine content, and (3) autoradiography with [^3^H]raclopride, [^3^H]DTBZ, [^3^H]GBR12935, [^3^H]PK11195, and [^3^H]UCB-J to measure binding at D2/3 receptors, vesicular monoamine transporter 2, dopamine transporters, mitochondrial translocator protein, and SV2A, respectively. rAAV-α-synuclein induced motor asymmetry and reduced tyrosine hydroxylase mRNA and dopamine content in ipsilateral brain regions. This was paralleled by elevated ipsilateral postsynaptic dopamine D2/3 receptor expression and immune activation, with no changes to synaptic SV2A density. In conclusion, α-synuclein overexpression results in dopaminergic degeneration that induced compensatory increases in D2/3 binding and immune activation, recapitulating many of the pathological characteristics of PD.

## 1. Introduction

Parkinson’s disease (PD) is a chronic neurodegenerative disease with symptoms of bradykinesia, rigidity and tremor, among others. It is characterized by progressive degeneration of the dopaminergic neurons in the substantia nigra (SN) and the presence of α-synuclein (ASYN) containing Lewy bodies [1]. ASYN is a 140 amino acid protein localized in the synapse, where it associates with vesicles. In a healthy brain, ASYN is present as a soluble unfolded monomer or helically folded tetramer [2,3], but during PD, ASYN aggregates and forms intraneuronal Lewy bodies and neurites [4,5,6]. Mutations and multiplications of the ASYN gene are found in families with PD, indicating the importance of ASYN in the disease [7].

It is estimated that clinical Parkinsonism occurs when 50% of dopamine terminal function is lost in the posterior putamen [8]. This can be examined using Positron Emission Tomography (PET) imaging, where PD patients exhibit reductions in putamen dopamine transporter (DAT) and vesicular monoamine transporter 2 (VMAT2) binding, compared to healthy individuals [9,10]. Furthermore, the dopamine deficiency can result in a compensatory upregulation of dopamine 2/3 (D2/3) receptors [11,12]. Dopaminergic deficits in PD are accompanied by neuroinflammation in the form of astrogliosis and microglial activation and proliferation that leads to expression of various proteins, including major histocompatibility complex class II (MHCII) and the 18 kDA translocator protein (TSPO) [13,14]. TSPO is a cholesterol and anion transporter generally expressed by activated microglia, as well as by other immune involved cells, such as astrocytes, at low levels, but can be upregulated in response to brain injuries and some neurological disorders [15,16]. Brain immune activation has been imaged with the TSPO PET radioligand, [^11^C]PK11195, where results show immune activation in the brain of PD patients, especially in the SN, the putamen, and cortical regions [17,18,19]. More recently, other microglial tracers have become available including [^11^C]PBR28, [^11^C]DPA-713 and [^18^F]FEPPA that have been applied to PD patients [19,20,21,22,23].

We have reported that the rodent model of PD induced by human ASYN overexpression using recombinant adeno-associated viral (rAAV) vectors, showed a decreased VMAT2 signal by PET imaging, suggesting terminal dysfunction that was associated to pathological ASYN aggregation in the striatal axons, in the absence of SN dopaminergic cell death. The pathology achieved was sufficient to induce motor defects, and we claimed this model to represent early PD [24]. In the current study, using the same animal model, we confirmed the effects on the presynaptic dopamine system. We examined whether the ASYN overexpression was limited to the dopaminergic terminals or if it caused a general loss of synaptic density by using the UCB-J marker of presynaptic synaptic vesicle glycoprotein 2A (SV2A) density. We tested the hypothesis that decreases in dopamine, measured by nigral tyrosine hydroxylase (TH) expression and striatal dopamine content, may result in compensatory increased postsynaptic striatal dopamine D2/3 receptor binding and neuroinflammation, even at early stages of PD-like neurodegeneration.

## 2. Materials and Methods

### 2.1. Animals and Stereotaxic Surgery

**Figure 1:**
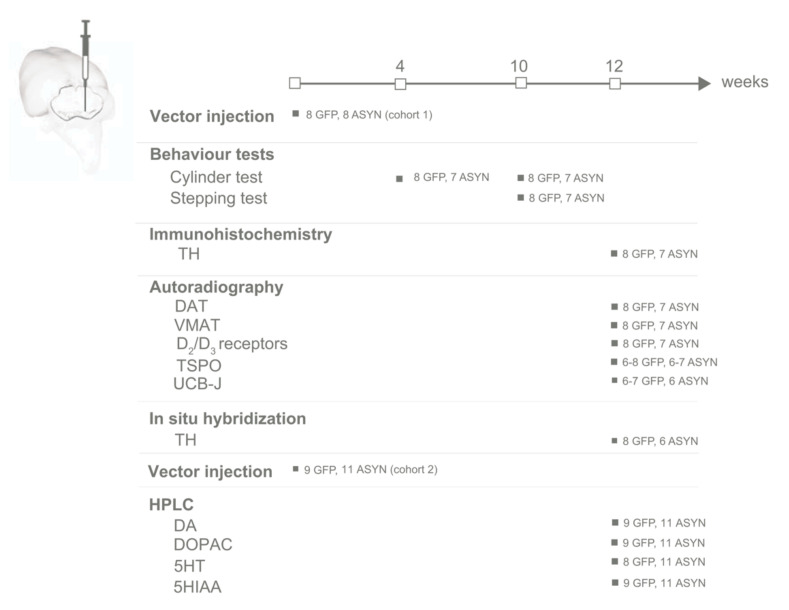
Timeline of studies. Two rat cohorts were injected with rAAV, containing either ASYN or GFP. In cohort 1 (*n* = 8 per group), behavioral tests were conducted at 4- and 10-weeks post-injection, and post-mortem immunohistochemistry and autoradiography at week 12 post-surgery. Cohort 2 (11 ASYN and 9 GFP) was used for HPLC studies.

Thirty-six female Sprague Dawley rats (225–250 g, *n* = 16 Taconic cohort 1, and *n* = 20 Janvier cohort 2, Denmark) were housed in pairs under a 12 h light/12 h dark cycle at an average of 21 °C and 55% humidity, with ad libitum access to food and water. We conducted experiments under humane conditions with ethical approval and in accordance with guidelines established by the Danish Animal Experiments Inspectorate (license 2017-15-0201-01295) and the European Legislation for the protection of vertebrate animals and in compliance with ARRIVE guidelines. Females were chosen due to the smaller sizer that allowed for grouped housing during long term studies. Cohort 1 was used for non-pharmacological behavioural studies, postmortem histology, autoradiography, and in situ hybridization. Cohort 2 was used for biochemical analysis of the striatal dopamine content. A timeline of the studies can be found in Figure 1.

Rats were numbered and pseudo-randomly allocated into an experimental or a control group and injected with rAAV containing either human wildtype ASYN or green fluorescent protein (GFP), respectively. The transgenes were expressed under the synapsin-1 promoter and enhanced using woodchuck hepatitis virus post-transcriptional regulatory element. Final stock for both vectors was 1 × 10^13^ genome copies/ml (VectorBiolabs, Malvern, PA, USA). Rats were anesthetized with medetomidine hydrochloride (0.67 mg/kg) and fentanyl (0.4 mg/kg) i.p. and placed in a stereotaxic frame (Stoelting, Wood Dale, IL, USA) with the nose bar set at −3.3. We injected 2 µL of the rAAV2/6 vector containing human wildtype ASYN (*n* = 8, cohort 1; *n* = 11, cohort 2) or green fluorescent protein (GFP) (*n* = 8 cohort 1; *n* = 9, cohort 2) into the right SN (−5.2 mm antero-posterior, 2.0 mm lateral to bregma, −7.2 mm dorso-ventral from dura) using a 5 μl Hamilton syringe fitted with a glass capillary (outer diameter of 60–80 μm). The volume was injected at a rate of 0.2 µL/30 s and the canula was kept at the target position for 5 additional min before slow retraction.

### 2.2. Motor Behavioural Tests, Cohort 1

All behavioural studies were conducted during the daylight period and evaluated by an experimenter blinded to the treatment groups. Forelimb preference was assessed using the cylinder test at 4 and 10 weeks post-rAAV-injection [25]. Rats were placed in a transparent cylinder and video was recorded for a minimum of 20 paw touches (3 min). Data was analyzed in slow motion using the software VCL Media Player and was presented as a percentage of the contralateral forelimb use compared to total number of touches. Forelimb akinesia was evaluated using the stepping test 10 weeks post-rAAV-injection [26]. The experimenter held the rat with both hind limbs plus one forelimb gently restrained in a fixed position, while allowing the unrestrained forelimb to touch the table. While moving the rat along the 90 cm table, the number of adjusting steps both in the forehand (i.e., the animal is moved left when the right paw touches the table and vice versa) and backhand direction (i.e., the animal is moved right when the right paw touches the table and vice versa) was counted. The backhand stepping scores served as the control.

### 2.3. Tissue Processing

Twelve weeks post-surgery, rats were decapitated, brains rapidly removed and frozen with isopentane cooled with dry ice, and stored at −80 °C. For autoradiography, brains (Cohort 1) were sectioned using a cryostat (HM 500 OM, MICROM International, GmbH, Walldorf, Germany) and 20 µm sections were mounted on poly-lysine-coated slides. For biochemical analysis (Cohort 2) brains were dissected, and striatal punches were processed using HPLC.

### 2.4. Immunohistochemistry, Cohort 1

To confirm successful surgery and transgene expression in the nigrostriatal pathway we performed immunohistochemistry on fresh-frozen slides using human ASYN (epitope 118–123, rabbit, 1:4000) and anti-GFP (rabbit, 1:200) (Abcam, Cambridge, UK) antibodies [24].

### 2.5. Autoradiography, Cohort 1

In order to study dopamine receptor and transporter binding, TSPO levels as a marker of immune activation, and SV2A synaptic density, an experimenter blinded to the treatment groups performed each tracer experiment using three consecutive slides containing four striatal sections per slide per brain region. We selected two slides for total binding and one for nonspecific binding and performed autoradiography according to previously described protocols [24,27]. Note that the VMAT2 data at the rostral level was used to confirm PET imaging data in another article published previously [24].

For [^3^H]GBR12935 binding to DAT, we incubated slides for 20 h at 4 °C in a buffer containing 50 mM Tris-HCl, 300 mm NaCl, 0.2% bovine serum albumin (pH 7.4) with 2 nM [^3^H]GBR12935 (Perkin Elmer, Skovlunde, Denmark), and 1 µM cis-flupentixol. [^3^H]GBR12935 labels the dopamine uptake sites on DAT and piperazine sites, and the presence of cis-flupentixol prevents piperazine labeling. Non-specific binding was achieved by adding 1 µM of unlabeled GBR12909, which binds to the same dopamine uptake site as GBR12935. For [^3^H]DTBZ binding to VMAT2, we pre-incubated slides for 25 min in 40 mM Tris-HCl buffer (pH 8.2). We determined the total binding by incubation with [^3^H]DTBZ (Biotrend Chemikalien GmbH, Cologne, Germany) at a final concentration of 7 nM in the same buffer for 90 min. Non-specific binding was measured in the presence of 1 µM unlabeled DTBZ. For [^3^H]Raclopride binding to D2/D3 receptors, we pre-incubated slides for 20 min in a buffer containing 50 mM Tris-HCl and 150 mM NaCl (pH 7.4), prior to a 45 min incubation in the same buffer containing 0.1% ascorbic acid and 2 nM [^3^H]Raclopride (Perkin Elmer, Skovlunde, Denmark). We assessed non-specific binding using 10 µM butaclamol, a D2 receptor antagonist. For [^3^H]PK11195 binding to the TSPO-site of activated microglia, we pre-incubated slides for 15 min in 50 mM Tris-HCl buffer (pH 7.4) and then incubated them in the same buffer in the presence of 1 nM [^3^H]PK11195 (Perkin Elmer, Skovlunde, Denmark) for 30 min. We used 20 µM of unlabelled PK11195 to assess nonspecific binding. After each tracer incubation, we washed the slides in a cold buffer, dipped them in cold, distilled water and dried them under a stream of cool air. We then placed them in a vacuum desiccator overnight.

Dried slides were then placed on Fuji imaging plates (BAS TR2025, Fujifilm, Saltsjö Boo, Sweden) for 7 days with a [^3^H] microscale (American Radiolabeled Chemicals, St. Louis, MO, USA). After exposure, we scanned the plates (BAS-5000, Fujifilm, Tokyo, Japan) and analysed the data using ImageGauge 4.03. An experimenter blinded to the treatment groups outlined the striatal or SN region on each section. We obtained total binding values as photostimulated luminescence values/mm^2^ and subtracted background values from every section. We then subtracted non-specific binding from total binding to obtain the specific binding (expressed as a % of internal control = (specific binding in the ipsilateral side)/(specific binding in the contralateral side) × 100). We calibrated binding data using a standard curve generated using the [^3^H]microscale.

For [^3^H]UCB-J binding to SV2A, we preincubated slides for 5 min in a buffer containing 50 mm Tris-HCl before incubating for 60 min in the same buffer with 1 nM [^3^H]UCB-J (Novandi chemistry AB, Södertälje, Sweden). Non-specific binding was achieved by adding 600 µM levetiracetam, which binds to the same binding site, thereby preventing [^3^H]UCB-J labeling. Slides were post-washed for 1 min in the buffer, dipped briefly three times in Milli-Q water, air dried, and read for 2 hours using BeaQuant v.1.14 (ai4r, Nantes, France) along with an in-house [^3^H] standard with known radioactive concentrations. Analysis was performed using Beamage v.2.1.7 software, and specific binding was obtained as described above.

### 2.6. In Situ Hybridization, Cohort 1

We performed in situ hybridization, as previously described [28]. We dried sections on slides which contained four coronal sections per rat at bregma: −5.8 (GFP *n* = 8, ASYN *n* = 6) before fixing the tissue in 4% paraformaldehyde for 5 min and rinsing in PBS twice for 2.5 min. We then acetylated sections in 0.25% acetic anhydride for 10 min. After this, we dehydrated sections in 70% ethanol for 5 min, 95% ethanol for 1 min, and 100% ethanol for 1 min. This was followed by delipidation in chloroform for 5 min, 100% ethanol for 1 min, and 95% ethanol for 1 min before they were left to dry. We visualized TH using a synthetic DNA antisense (Eurofins Genomics, Ebersberg, Germany) for TH mRNA (probe sequence: 5′-GGAATTGGTTCACCGTGCTTGTACTGGAAGGCA-3′). We diluted the probe to 5 pmol/µL using ultra-pure distilled water and labelled it with [α^35^S]dATP (1250 Ci/mmol, PerkinElmer, Boston, USA) using terminal transferase (Roche, Mannheim, Germany). We diluted 10 µL labelled probe in 1 ml hybridization buffer (buffer preparation: 450 ml distilled water, 400 ml 20X SCC (SCC: 175.3 g NaCl and 88.2 g trisodiumcitrate in 1 L distilled water), 100 ml ≥99.5% formamide, 20 g dextran sulfate (Sigma), 4 ml Denhardt’s solution 50 × (Sigma), 1 ml tRNA (Roche, Mannheim, Germany), and 10 ml deoxyribonucleic acid single stranded from salmon sperm (Sigma)) and 10 µL 1M dithiothretiol. We added hybridization solution to each slide overnight in a 37 °C humid chamber to hybridize. We then washed slides in 1X SCC at 60 °C for 2 × 15 min, 30 min at 60 °C, and 30 min at room temperature, and dipped them briefly in deionized water, followed by 1 min in 70% ethanol, and 1 min in 95% ethanol, before drying. The slides were placed on an imaging plate (BAS MS2040, Fujifilm, Saltsjö Boo, Sweden) along with a [^14^C] standard and exposed for two weeks before processing on an imaging plate scanner (Fujifilm). The digital images obtained were used to manually draw the regions of interest (Image Gauge) and for analysis by an experimenter blinded to the treatment groups.

### 2.7. HPLC, Cohort 2

Striatal punches were dissected, starting at bregma: 1.4, two overlapping punches (1.5 mm in diameter, 1.8 mm deep) in the center of the striatum, GFP *n* = 9, ASYN *n* = 11. We examined dopamine (DA), dihydroxyphenylacetic acid (DOPAC), serotonin (5HT), and 5-hydroxyindoleacetic acid (5-HIAA) in the striatal punches using HPLC as previously described [29]. Briefly, we homogenized samples by sonication in HClO_4_ and filtered through Costar cellulose acetate filter tubes (0.22 μm; Corning Inc., Corning, NY, USA) and then separated DA, DOPAC, 5-HIAA, and 5HT on a 150 × 3 mm Hypersil™ BDS C18 3 μm particle column kept at 28 °C. To carry out detection, we used a Thermo Scientific™ Dionex™ model 6011RS ultra Coulometric Analytical cell (E1: −150 mV: E2: +250 mV vs Pd reference) attached to a Thermo Scientific Dionex Ultimate 3000 UHPLC system while eluting the analytes with a MDTM mobile phase (Thermo Scientific™ Dionex™ Test Phase, 70-3829) at a flow rate of 0.5 ml/min.

### 2.8. Statistics

We selected group sizes of at least 8 since we have previously been able to detect statistically significant differences in our autoradiography studies using *n* = 6. We used a GraphPad Prism (v. 6.0) for the statistical analysis. We used two-way ANOVA with Tukey’s post-hoc test for multiple comparisons for the cylinder test, and one-tailed unpaired t-test for the stepping test. For the autoradiography analysis, we used unpaired t-test to determine differences in specific binding among groups (% change from injected to non-injected side). We confirmed normality using a Shapiro-Wilk test before performing each t-test and used an F-test to assess if variances of the groups were significantly different. If so, we performed unpaired t-tests with Welch’s correction.

Data will be made available upon reasonable request to the corresponding author.

## 3. Results

### 3.1. Transgene Expression

In order to evaluate transgene expression and location of our injections, we performed histology on fresh frozen sections. The use of fresh-frozen slides did not give quality sufficient for detailed microscopic imaging but could be used to confirm transgene expression in ventral midbrain and striatum, thus controlling injection placement and viral transduction. We found homogenous GFP or ASYN immunostaining in the ipsilateral side of the ventral midbrain and striatum of all except two ASYN-injected animals (Supplemental Figure S1). One animal showed immunostaining against ASYN limited to the lateral striatum (Supplemental Figure S1C), versus the more homogeneous expression in the striatum observed in the others (Supplemental Figure S1B). We included this animal in the analyses as it showed robust immunostaining for ASYN in the SN, indicating accurate injection. We, however, excluded another animal in the ASYN group due to a potential failed injection, as ASYN immunostaining was not detected (Supplemental Figure S1D). For the final analysis, our groups in cohort 1 consisted of 8 GFP-injected animals and 7 ASYN-injected animals (Figure 1).

### 3.2. Behavioural Tests

**Figure 2:**
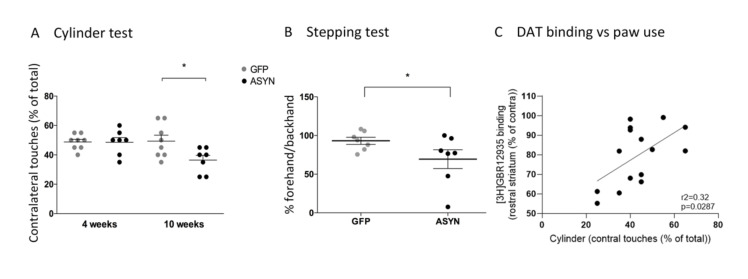
(**A**) The cylinder test was performed at 4 and 10 weeks after the injections and contralateral (left) forelimb touches were measured as percent of total touches in GFP (grey circles) and ASYN (black circles) injected rats. (**B**) The stepping test was measured as percent forehand/backhand touches in the GFP and ASYN expressing animals. Data are represented as mean ± SEM, * *p* < 0.05. A two-way ANOVA with Tukey’s post-hoc test was used for the cylinder test, and one-tailed unpaired t-test for the stepping test. (**C**) Pearson correlation between rostral striatal GBR12935 binding to DAT and contralateral paw use in the cylinder test. *N* = 7–8 animals per group.

To evaluate whether ASYN overexpression in our model led to motor impairment, we tested motor asymmetry using two spontaneous behavioural tests which did not rely on pharmacological manipulation. In the cylinder test, no paw bias was seen 4 weeks after rAAV2/6 injection, however, after 10 weeks the ASYN group showed a significant decrease in the use of the contralateral paw compared to the GFP animals (Figure 2A). ASYN rats also displayed significant akinesia of the contralateral paw in the stepping test as the number of steps in the backhand direction were significantly decreased when compared to GFP rats (Figure 2B). Thus, our data revealed that prolonged unilateral ASYN expression in the nigrostriatal system resulted in motor asymmetry (Figure 2).

### 3.3. ASYN Expression Induced Reduction in the Nigral TH Expression and Striatal Dopamine Levels

**Figure 3:**
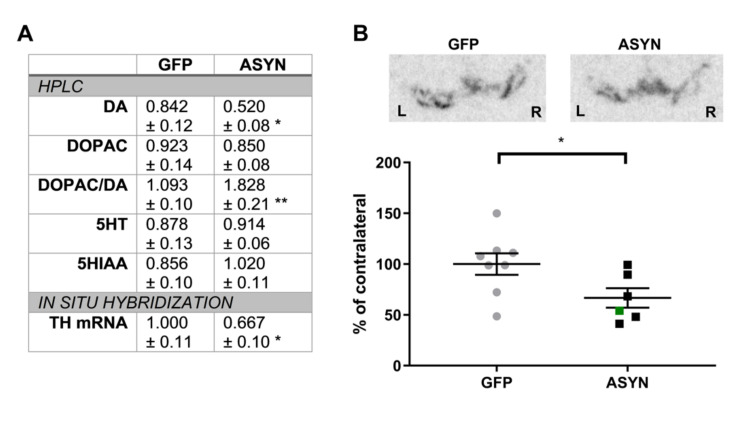
(**A**) HPLC measurements in striatum and in situ hybridization in SN. Data are presented as ipsilateral/contralateral ratio. In situ hybridization: GFP (*n* = 8), ASYN (*n* = 6), HPLC: GFP (*n* = 9 (*n* = 8 for 5HT)), ASYN (*n* = 11). (**B**) Representative in situ hybridization of TH mRNA in the SN. The graph at the bottom shows the ipsilateral value as a percentage of the contralateral binding. GFP (*n* = 8), ASYN (*n* = 6). The green square represents the animal with lateral ASYN expression in the striatum. Distance from bregma −5.52 mm. Data are mean ± SEM, * *p* < 0.05, ** *p* < 0.01 (unpaired t-tests).

To evaluate the integrity of the dopaminergic system in striatum, we analyzed the content of dopamine and its metabolites by HPLC in striatal punches 12 weeks post-surgery (Figure 3A). While the level of dopamine in the ipsilateral striatum was 84% of that seen in the contralateral striatum in GFP animals, ASYN overexpression resulted in significantly lower content of dopamine in the ipsilateral (52%) vs. contralateral side, indicating a loss of terminals or a downregulation of the dopamine synthesis (*p* < 0.05). Although no difference was found in the dopamine metabolite DOPAC among the groups, we observed a significant increase in the DOPAC/dopamine ratio, a measure of dopamine turnover, in the ASYN animals compared to the GFP animals. No significant differences were found in 5HT or 5HIAA levels, confirming a selective dopaminergic neurodegeneration.

We assessed the nigral dopaminergic neuronal population by analyzing the level of TH mRNA in neurons of the SN compacta (Figure 3A,B). The amount of [α^35^S]dATP-labelled tracer binding to TH mRNA in the SN was similar in both sides of the SN in GFP overexpressing animals. However, ASYN overexpression resulted in a decrease of the TH mRNA signal in the ipsilateral SN. Thus, ASYN overexpression induced a decrease in TH expression, suggesting down-regulation of TH or dopaminergic neuronal loss.

### 3.4. ASYN Overexpression in Nigrostriatal Neurons Induced Pre-and Post-Synaptic Changes in Striatum

**Figure 4:**
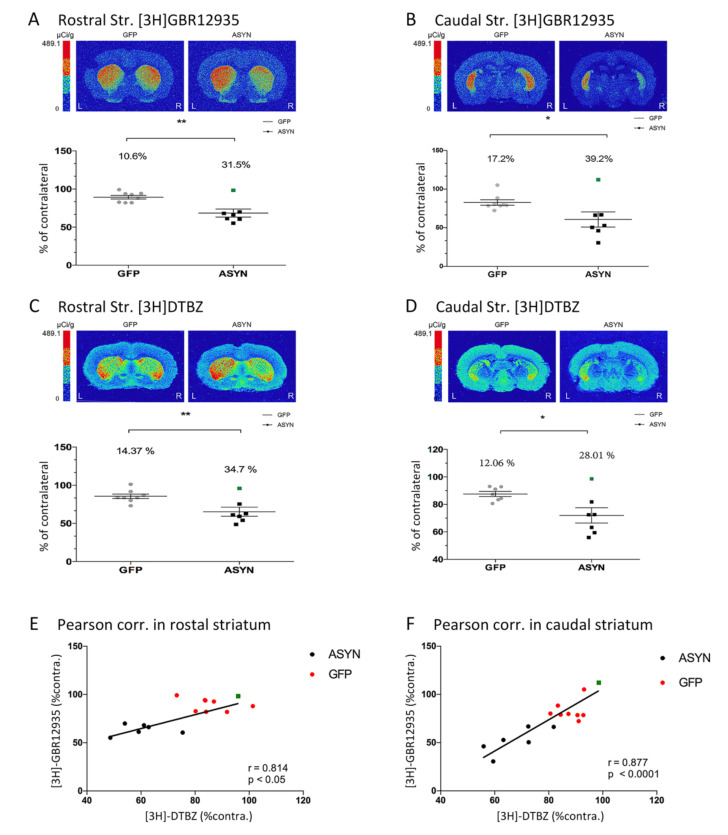
Representative autoradiograms of [^3^H]GBR12935 binding to DAT in the (**A**) rostral and (**B**) caudal striatum and of [^3^H]DTBZ binding to VMAT2 in the (**C**) rostral and (**D**) caudal striatum. Graphs in each panel show the ipsilateral value as percentage of the contralateral binding. The green square represents the animal with lateral ASYN expression in the striatum. GFP (*n* = 8), ASYN (*n* = 7). The percentage shows the % decrease in ipsilateral binding, compared to contralateral binding. Data are mean ± SEM, * *p* < 0.05, ** *p* < 0.01 (unpaired t-tests with Welch’s correction). Distance from bregma 0.48 mm, −1.80 mm, −0.26 mm, and −0.92 mm, in (**A**–**D**), respectively. Pearson correlations, with r and *p* value indicated, of DAT and VMAT2 are presented in (**E**) the rostral, and (**F**) the caudal level of the striatum.

In order to analyze the consequences of transgene expression in the pre- and post-synaptic striatal dopaminergic system, we compared the binding signal of the different dopaminergic ligands at striatal level. Here we confirmed and extended the studies presented in Phan et al., in which VMAT2 autoradiography at the rostral striatum was performed, along with DAT immunohistochemistry [24], by applying radioligands for DAT, VMAT2, and D2/3 receptors to sections from both the caudal and rostral striatum. Our analysis revealed that [^3^H]GBR12935 binding to DAT was significantly decreased in the ipsilateral striatum in the ASYN group, compared to the GFP group with a 31.5% decrease of [^3^H]GBR12935 binding in the ipsilateral rostral striatum (Figure 4A) and 39.2% in the caudal striatum, compared to the contralateral side (Figure 4B). When comparing the motor behavior and the three markers analyzed, we found a significant (*p* = 0.029, r^2^ = 0.32), although weak, positive correlation between the rostral striatal DAT binding (% of contra) and the use of the contralateral paw in the cylinder test at 10 weeks (Figure 2C). This suggested that the dopaminergic axonal degeneration was associated with the motor deficits.

DTBZ binding to VMAT2 was decreased in the ipsilateral vs contralateral rostral striatum by 34.7% (Figure 4C) and by 28% in the caudal striatum (Figure 4D) in response to ASYN overexpression. Similar to the DAT tracer findings, significantly smaller decreases were observed in DTBZ binding in the GFP group. Note that the ASYN animal that showed lateral expression of ASYN (Supplemental Figure S1C) had smaller changes in % internal control when compared to the rest of the ASYN injected animals (Figure 4A–D, green squares). Direct comparison of the percentage reduction of DAT and VMAT expression in all animals showed significant positive correlations in both the rostral (*p* < 0.05) (Figure 4E) and caudal (*p* < 0.0001) striatum (Figure 4F).

**Figure 5:**
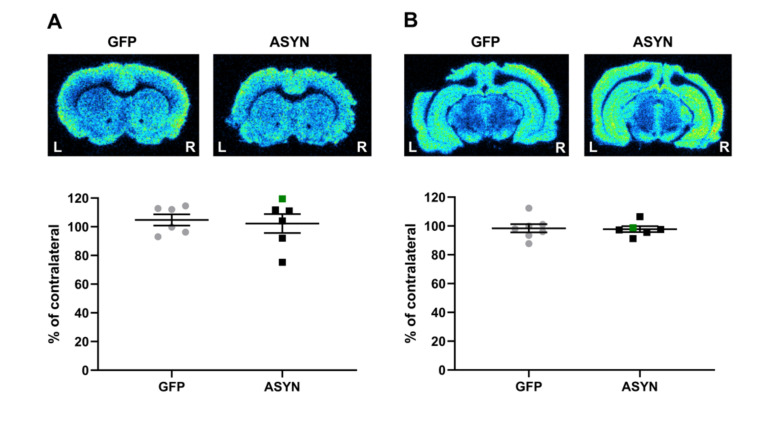
Representative autoradiograms of [^3^H]UCB-J binding to SV2A in (**A**) the striatum and (**B**) SN. Graphs at the bottom of each panel show the ipsilateral value as percentage of the contralateral binding. The green square represents the rat with lateral ASYN expression in the striatum. Striatum: GFP (*n* = 6), ASYN (*n* = 7), SN: GFP (*n* = 7), ASYN (*n* = 7). Data are mean ± SEM. Distance from bregma 0.48 mm and −5.8 mm in (**A**,**B**), respectively.

To examine whether this loss of VMAT positive presynaptic terminals corresponded to a decrease of presynaptic density, we performed [^3^H]UCB-J autoradiography at the striatal and nigral level (Figure 5). No differences in [^3^H]UCB-J binding to SV2A were observed, indicating that the loss of dopaminergic presynaptic terminals was not sufficient to induce a significant decrease in the overall synaptic density in the striatum.

**Figure 6:**
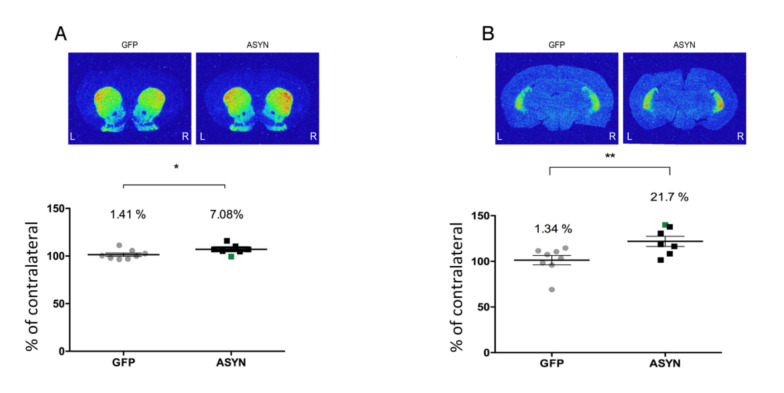
Representative autoradiograms of [^3^H]raclopride binding to D2/3 receptors in the (**A**) rostral and (**B**) caudal striatum. Graphs at the bottom of each panel show the ipsilateral value as percentage of the contralateral binding. The green square represents the animal with lateral ASYN expression in the striatum. GFP (*n* = 8), ASYN (*n* = 7). Data are mean ± SEM, * *p* < 0.05, ** *p* < 0.01 (unpaired t-tests). Distance from bregma 1.20 mm and −2.80 mm in (**A**,**B**), respectively.

To evaluate whether the ASYN induced dopaminergic degeneration led to changes on dopamine D2/3 receptors in the post-synaptic neurons, we performed autoradiography studies with [^3^H]raclopride. ASYN animals showed a significant increase in [^3^H]raclopride binding to D2/3 receptors in the ipsilateral vs contralateral striatum both in the rostral by 7% (Figure 6A) and caudal striatum by 22% (Figure 6B), suggesting a compensatory increase of D2/3 receptors induced by the long-term expression of ASYN. These changes in ASYN-injected animals were significantly larger than those in the GFP group at both levels of the striatum (Figure 6).

### 3.5. ASYN Overexpression Induced Immune Activation in SN and Striatum

**Figure 7:**
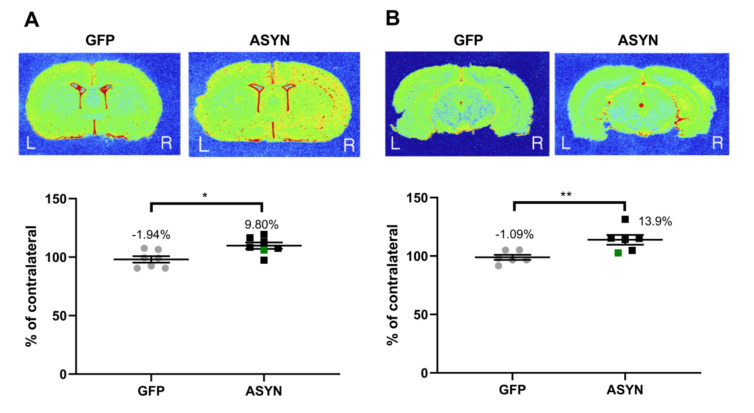
Representative autoradiograms of [^3^H]PK11195 binding to TSPO in the (**A**) striatum and (**B**) SN. Graphs at the bottom of each panel show the ipsilateral value as percentage of the contralateral binding. The green square represents the animal with lateral ASYN expression in the striatum. SN: GFP (*n* = 6), ASYN (*n* = 6), Striatum: GFP (*n* = 8), ASYN (*n* = 7). We present the data as mean ± SEM, * *p* < 0.05, ** *p* < 0.01 (unpaired t-tests). Distance from bregma −5.8 and −6.04 for SN levels ((**A**), left and right, respectively) and 0.2 mm for striatum (**B**).

To study immune activation, we applied the [^3^H]PK11195 tracer to brain sections at the level of the SN and striatum. We found a 9.8% increase in [^3^H]PK11195 binding to the TSPO in the ipsilateral vs contralateral sides of the striatum (Figure 7A) and a 13.9% increase in the SN (Figure 7B) of the ASYN group, which were significantly higher than the changes observed in the GFP-injected animals. The SN and striatum data from one animal in the GFP group was excluded as an outlier using Grubbs’ outlier test, and the SN data from one rat in each group was excluded due to damage to the slides during the autoradiography procedure. During the analysis, we noted that the binding values in the contralateral SN were significantly increased by 26% in the SN in the ASYN group vs the GFP group (*p* < 0.05), which suggested bilateral microglia activation, an effect not present in the striatal data.

## 4. Discussion

Here we showed that the overexpression of human rAAV2/6-ASYN led to motor deficits, induced changes in the pre- and postsynaptic dopaminergic system, and promoted neuroinflammation measured by TSPO expression in the striatum and SN. In our previous work, we showed that the intranigral injection of this rAAV2/6-ASYN resulted in a rat PD model which displayed terminal dysfunction and pathological ASYN aggregation at the level of the striatum, in the absence of significant SN dopaminergic cell death [24]. Our current study further extended those observations and confirmed that ASYN overexpression induced changes in dopaminergic nigrostriatal system as shown by the reduced TH expression in SN and striatal dopamine levels, that paralleled motor defects. We confirmed that synaptic changes occurred in dopaminergic axons, as shown by the decreased DAT and VMAT2 binding in striatum. These changes in the dopaminergic neurotransmission induced a compensatory increase of the D2/D3 postsynaptic binding. Moreover, the ASYN induced degenerative process was associated with immune activation in basal ganglia as shown by the increased TSPO binding in SN and striatum, with no clear effect on SV2A density.

The ASYN overexpression induced a loss of axonal pre-synaptic markers, VMAT2 and DAT, and, through a compensatory mechanism, an increased D2/3 receptor expression. This is likely due to the D2/3 receptors sensitivity to endogenous levels of dopamine and that led to an upregulation in response to lower dopamine levels in the synaptic cleft. This agreed with prior data in PD patients and other PD models. Indeed, PD patients exhibited reductions in putamen DAT and VMAT2 binding compared to healthy individuals and a compensatory upregulation of dopamine D2/3 receptors [9,10,11,12]. Chronic low-dose 1-methyl-4-phenyl-1,2,3,6-tetrahydropyridine in non-human primates resulted in decreased binding in DAT and VMAT2 but without any change in D2/3 receptors [30]. ASYN rodent models have also shown decreased DAT binding. For instance, transgenic rats expressing human mutant A53T had a progressive decrease in DAT as they aged from 4 to 16 months [31]. Progressive decrease of DAT binding using PET has also been shown in the human A53T ASYN rAAV2/7 rat PD model [32]. The upregulation of D2 receptors has also previously been observed in genetic PD models, such as that observed using autoradiography in the striatum of the DJ-1 and PINK knockouts [33]. Acute intranigral injections of nitrated recombinant ASYN in rats led to dopaminergic neuronal death in SN and down-regulation of D2 receptors in striatum [34]. However, to our knowledge, we are the first to show a significant upregulation of D2 receptors in a PD model based on long-lasting overexpression of human ASYN in rats.

We sought to test whether the ASYN overexpression in dopaminergic terminals resulted in significant overall decrease of striatal synaptic terminals. To do so, we used [^11^C]UCB-J, an SV2A ligand. PD patients showed reduced UCBJ binding in the SN, red nucleus, locus coeruleus, caudate, putamen, brainstem, and thalamus [35,36,37,38]. We have recently shown decreased synaptic density using the acute 6-OHDA PD model (6.2%) [39] and in a chronic ASYN-based model induced by unilateral striatal injection of ASYN fibrils (2.5–5.7%) [40]. Here, however, we did not observe any changes in presynaptic density. The changes found in the previous studies were of small magnitude, consistent with views that nigral dopaminergic projections constitute only 10% of terminals in the striatum [41,42]. The lack of a change found in the current model may be due to the mild neuronal loss [24] vs. the significant loss observed in the ASYN fibril PD model (64.2% (15 weeks post-surgery)–56.3% (22 weeks post-surgery) loss of TH+ neurons in the ipsilateral SN (compared to the contralateral SN) [40]). One cannot exclude that changes in SV2A might be apparent at later timepoints after rAAV injections, and further studies are warranted for clarification.

The ASYN overexpression resulted in a significant motor deficit that was apparent in the contralateral paw with both tests used after 10 weeks. This was related to the axonal dopaminergic neurodegeneration as shown by the significant correlation between the paw use in the cylinder test and the rostral striatal DAT binding. Accordingly, lower ipsilateral axonal DAT binding correlated with decreased use of the contralateral paw in favor of the ipsilateral one. We also found a significant correlation between binding levels of VMAT2 and DAT at both the rostral and caudal levels of the striatum. This association is similar to that previously described between [^11^C]DTBZ and optical density DAT values in a 6-OHDA rat PD model [43]. Interestingly, the caudal level of the striatum had the strongest correlation, which might be explained by the ASYN induced axonal degeneration, which as we described previously in this model, was more prominent in the caudal striatum compared to the rostral part [24]. Such caudo-rostral susceptibility was also observed in other models and in PD patients [44,45]. Accordingly, and as a compensatory mechanism, the observed increase in D2/3 receptors exhibited a rostral to caudal gradient, with highest expression of D2/3 receptors in the caudal part of the striatum. This was in agreement with human studies in early PD demonstrating increased D2/3 receptors in the putamen, while the caudate remained intact [12]. Moreover, this was also consistent with the rostral-caudal down-regulation of TH axonal expression in the striatum that we have previously described in the model [24]. The decreased TH expression could be a result of the decrease in nigral TH mRNA expression we observed in the current study. This gradient was, however, not observed for VMAT2 and DAT binding with similar declines observed in the rostral and caudal striatum. We suggested that the D2/3 receptors may be upregulated as a response to the ASYN mediated downregulation of TH and decreased synaptic dopamine content, and not necessarily due to the loss of the dopaminergic terminals. In fact, our HPLC analysis showed a significant decrease in dopamine that was of greater magnitude than the VMAT and DAT decreases, suggesting a downregulation of the synthesis of dopamine in surviving axons, and an increased dopamine turnover as shown by the higher DOPAC/dopamine ratio. Accordingly, an early progressive decline in dopamine release prior to SN neuronal loss has been described in the model, where a decrease of 50% was observed 10 days after the rAAV injections and before any cell death was found [46]. The decrease of dopamine was also supported by the significant motor asymmetry observed in the rats. Our data also suggested that a threshold of neuronal overexpression of ASYN must be reached to cause significant changes in the pre-and postsynaptic dopaminergic system, as the animal that only expressed ASYN in the lateral part of the striatum, did not show alterations in the pre- and postsynaptic dopaminergic system compared to the rest of animals with homogenous expression in the nigrostriatal system.

Neuroinflammation is a characteristic pathological finding in PD brains and the activation of microglia is correlated to ASYN deposition in brain [47]. In fact, there is a wealth of evidence suggesting that ASYN can act as a damage associated molecular pattern (DAMP) and induce microglia activation [48]. We detected significantly increased [^3^H]PK11195 binding in the ipsilateral striatum and SN of the ASYN expressing rats, which was not observed in the GFP group. These findings are consistent with prior studies by us and others in the rAAV-ASYN rat model using immunohistochemical markers of neuroinflammation and cytokine analysis [49,50]. They are also in line with the microgliosis we have previously shown in a similar rAAV-ASYN based PD model in non-human primates [51]. Furthermore, our data are in agreement with PET studies using [^11^C]PK11195 and other tracers of neuroinflammation showing increased immune activation in PD patients [17,18,19,23], post-mortem histological analysis [52], and in rats expressing rAAV-A53T-ASYN [53,54]. Unilateral increases in [^3^H]PK11195 autoradiographic binding were previously described in the SN and striatum of the 6-OHDA rat model while the neurodegenerative process occurred, but [^3^H]PK11195 binding returned to basal levels once the cell death reached its plateau [55]. In the ASYN overexpressing rats of this study, we observed a unilateral increase of TSPO signal in the ipsilateral side of the striatum and the SN, supporting an immune activation associated with ASYN induced pathology and neurodegeneration. Neuroinflammation has also been observed in other ASYN based models, such as transgenic ASYN mice [56,57], and in models induced by injection of ASYN fibrils [40,58,59]. Early immune activation in midbrain was associated with prodromal PD as increased [^11^C]PK11195 PET binding was found in midbrain of patients with REM sleep disorders, which have a high risk of developing PD [58]. The immune activation progresses to the putamen in diagnosed PD patients [17,59] that already exhibited motor defects. Our PK11195 autoradiography data also showed significantly increased immune activation in the contralateral side of the SN in the ASYN vs the GFP group, suggestive of a bilateral response. Indeed, our group has previously found bilaterally increased [^11^C]PK11195 binding in the midbrain of minipigs unilaterally injected with rAAV vectors containing ASYN [60]. This might be a consequence of a generalized immune activation, by cytokine or cellular interaction; indeed, peripheral immune cells also partake in the immune response during PD [48]. However, it might also be due to cross-innervation of the contralateral regions, which may result in the immune activation in the contralateral hemisphere [61].

## 5. Conclusions

In conclusion, we demonstrate that unilateral overexpression of human ASYN in the nigrostriatal system leads to motor deficits and reduced TH mRNA in the SN, decreased levels of dopamine in the striatum and mild, yet significant, compensatory increased expression of striatal D2/3 receptors. We also show increased TSPO expression by autoradiography as a marker of neuroinflammation at the level of the striatum and SN. Our previous study supported the model as a useful tool to study early stages of PD [24]. In the current study, the lack of significant loss in SV2A, the small increase in raclopride binding to D2/3 receptors, and mild neuroinflammation, further confirms this as a model of early PD. These findings encourage the use of the rAAV2/6 ASYN model as a platform for the evaluation of neuronal changes and treatments at the early stage of PD.

## Data Availability

Data are available upon reasonable request to the corresponding author.

## Supplementary Materials

The following are available online at https://www.mdpi.com/article/10.3390/biomedicines9121876/s1, Figure S1: Immunohistochemistry of ASYN and GFP expressing animals.

## References

[1] Goedert M., Spillantini M.G., Del Tredici K., Braak H. (2013). 100 years of Lewy pathology. Nat. Rev. Neurol..

[2] Bartels T., Choi J.G., Selkoe D.J. (2011). Alpha-Synuclein occurs physiologically as a helically folded tetramer that resists aggregation. Nature.

[3] Dettmer U., Newman A.J., Luth E.S., Bartels T., Selkoe D. (2013). In vivo cross-linking reveals principally oligomeric forms of alpha-synuclein and beta-synuclein in neurons and non-neural cells. J. Biol. Chem..

[4] Spillantini M.G., Crowther R.A., Jakes R., Hasegawa M., Goedert M. (1998). α-Synuclein in filamentous inclusions of Lewy bodies from Parkinson’s disease and dementia with lewy bodies. Proc. Natl. Acad. Sci. USA.

[5] Bridi J.C., Hirth F. (2018). Mechanisms of alpha-Synuclein Induced Synaptopathy in Parkinson’s Disease. Front. Neurosci..

[6] Spillantini M.G., Goedert M. (2018). Neurodegeneration and the ordered assembly of alpha-synuclein. Cell Tissue Res..

[7] Singleton A.B., Farrer M., Johnson J., Singleton A., Hague S., Kachergus J., Hulihan M., Peuralinna T., Dutra A., Nussbaum R. (2003). alpha-Synuclein locus triplication causes Parkinson’s disease. Science.

[8] Morrish P.K., Rakshi J.S., Bailey D.L., Sawle G.V., Brooks D.J. (1998). Measuring the rate of progression and estimating the preclinical period of Parkinson’s disease with [18F]dopa PET. J. Neurol. Neurosurg. Psychiatry.

[9] Lee C.S., Samii A., Sossi V., Ruth T.J., Schulzer M., Holden J.E., Wudel J., Pal P.K., De la Fuente-Fernandez R., Calne D.B. (2000). In vivo positron emission tomographic evidence for compensatory changes in presynaptic dopaminergic nerve terminals in Parkinson’s disease. Ann. Neurol..

[10] Lin S.C., Lin K.J., Hsiao I.T., Hsieh C.J., Lin W.Y., Lu C.S., Wey S.P., Yen T.C., Kung M.P., Weng Y.H. (2014). In vivo detection of monoaminergic degeneration in early Parkinson disease by (18)F-9-fluoropropyl-(+)-dihydrotetrabenzazine PET. J. Nucl. Med..

[11] Antonini A., Schwarz J., Oertel W.H., Pogarell O., Leenders K.L. (1997). Long-term changes of striatal dopamine D2 receptors in patients with Parkinson’s disease: A study with positron emission tomography and [11C]raclopride. Mov. Disord..

[12] Rinne J.O., Laihinen A., Ruottinen H., Ruotsalainen U., Nagren K., Lehikoinen P., Oikonen V., Rinne U.K. (1995). Increased density of dopamine D2 receptors in the putamen, but not in the caudate nucleus in early Parkinson’s disease: A PET study with [11C]raclopride. J. Neurol. Sci..

[13] McGeer P.L., Itagaki S., Boyes B.E., McGeer E.G. (1988). Reactive microglia are positive for HLA-DR in the substantia nigra of Parkinson’s and Alzheimer’s disease brains. Neurology.

[14] Venneti S., Lopresti B.J., Wiley C.A. (2006). The peripheral benzodiazepine receptor (Translocator protein 18kDa) in microglia: From pathology to imaging. Prog. Neurobiol..

[15] Papadopoulos V., Baraldi M., Guilarte T.R., Knudsen T.B., Lacapere J.J., Lindemann P., Norenberg M.D., Nutt D., Weizman A., Zhang M.R. (2006). Translocator protein (18kDa): New nomenclature for the peripheral-type benzodiazepine receptor based on its structure and molecular function. Trends Pharmacol. Sci..

[16] Nutma E., Ceyzeriat K., Amor S., Tsartsalis S., Millet P., Owen D.R., Papadopoulos V., Tournier B.B. (2021). Cellular sources of TSPO expression in healthy and diseased brain. Eur. J. Nucl. Med. Mol. Imaging.

[17] Ouchi Y., Yoshikawa E., Sekine Y., Futatsubashi M., Kanno T., Ogusu T., Torizuka T. (2005). Microglial activation and dopamine terminal loss in early Parkinson’s disease. Ann. Neurol..

[18] Gerhard A., Pavese N., Hotton G., Turkheimer F., Es M., Hammers A., Eggert K., Oertel W., Banati R.B., Brooks D.J. (2006). In vivo imaging of microglial activation with [11C](R)-PK11195 PET in idiopathic Parkinson’s disease. Neurobiol. Dis..

[19] Gerhard A. (2016). TSPO imaging in parkinsonian disorders. Clin. Transl. Imaging.

[20] Varnas K., Cselenyi Z., Jucaite A., Halldin C., Svenningsson P., Farde L., Varrone A. (2019). PET imaging of [(11)C]PBR28 in Parkinson’s disease patients does not indicate increased binding to TSPO despite reduced dopamine transporter binding. Eur. J. Nucl. Med. Mol. Imaging.

[21] Terada T., Yokokura M., Yoshikawa E., Futatsubashi M., Kono S., Konishi T., Miyajima H., Hashizume T., Ouchi Y. (2016). Extrastriatal spreading of microglial activation in Parkinson’s disease: A positron emission tomography study. Ann. Nucl. Med..

[22] Ghadery C., Koshimori Y., Coakeley S., Harris M., Rusjan P., Kim J., Houle S., Strafella A.P. (2017). Microglial activation in Parkinson’s disease using [(18)F]-FEPPA. J. Neuroinflammation.

[23] Koshimori Y., Ko J.H., Mizrahi R., Rusjan P., Mabrouk R., Jacobs M.F., Christopher L., Hamani C., Lang A.E., Wilson A.A. (2015). Imaging Striatal Microglial Activation in Patients with Parkinson’s Disease. PLoS ONE.

[24] Phan J.A., Stokholm K., Zareba-Paslawska J., Jakobsen S., Vang K., Gjedde A., Landau A.M., Romero-Ramos M. (2017). Early synaptic dysfunction induced by alpha-synuclein in a rat model of Parkinson’s disease. Sci. Rep..

[25] Kirik D., Rosenblad C., Bjorklund A., Mandel R.J. (2000). Long-term rAAV-mediated gene transfer of GDNF in the rat Parkinson’s model: Intrastriatal but not intranigral transduction promotes functional regeneration in the lesioned nigrostriatal system. J. Neurosci..

[26] Olsson M., Nikkhah G., Bentlage C., Bjorklund A. (1995). Forelimb akinesia in the rat Parkinson model: Differential effects of dopamine agonists and nigral transplants as assessed by a new stepping test. J. Neurosci..

[27] Minuzzi L., Olsen A.K., Bender D., Arnfred S., Grant R., Danielsen E.H., Cumming P. (2006). Quantitative autoradiography of ligands for dopamine receptors and transporters in brain of Gottingen minipig: Comparison with results in vivo. Synapse.

[28] Christiansen S.H., Woldbye D.P. (2010). Regulation of the galanin system by repeated electroconvulsive seizures in mice. J. Neurosci. Res..

[29] Liebenberg N., Jensen E., Larsen E.R., Kousholt B.S., Pereira V.S., Fischer C.W., Wegener G. (2018). A Preclinical Study of Casein Glycomacropeptide as a Dietary Intervention for Acute Mania. Int. J. Neuropsychopharmacol..

[30] Chen M.K., Kuwabara H., Zhou Y., Adams R.J., Brasic J.R., McGlothan J.L., Verina T., Burton N.C., Alexander M., Kumar A. (2008). VMAT2 and dopamine neuron loss in a primate model of Parkinson’s disease. J. Neurochem..

[31] Yamasaki T., Fujinaga M., Kawamura K., Furutsuka K., Nengaki N., Shimoda Y., Shiomi S., Takei M., Hashimoto H., Yui J. (2016). Dynamic Changes in Striatal mGluR1 But Not mGluR5 during Pathological Progression of Parkinson’s Disease in Human Alpha-Synuclein A53T Transgenic Rats: A Multi-PET Imaging Study. J. Neurosci..

[32] Van der Perren A., Toelen J., Casteels C., Macchi F., Van Rompuy A.S., Sarre S., Casadei N., Nuber S., Himmelreich U., Osorio Garcia M.I. (2015). Longitudinal follow-up and characterization of a robust rat model for Parkinson’s disease based on overexpression of alpha-synuclein with adeno-associated viral vectors. Neurobiol. Aging.

[33] Sun J., Kouranova E., Cui X., Mach R.H., Xu J. (2013). Regulation of dopamine presynaptic markers and receptors in the striatum of DJ-1 and Pink1 knockout rats. Neurosci. Lett..

[34] Yu Z., Xu X., Xiang Z., Zhou J., Zhang Z., Hu C., He C. (2010). Nitrated alpha-synuclein induces the loss of dopaminergic neurons in the substantia nigra of rats. PLoS ONE.

[35] Cai Z., Li S., Matuskey D., Nabulsi N., Huang Y. (2019). PET imaging of synaptic density: A new tool for investigation of neuropsychiatric diseases. Neurosci. Lett..

[36] Delva A., Van Weehaeghe D., Koole M., Van Laere K., Vandenberghe W. (2020). Loss of Presynaptic Terminal Integrity in the Substantia Nigra in Early Parkinson’s Disease. Mov. Disord..

[37] Matuskey D., Tinaz S., Wilcox K.C., Naganawa M., Toyonaga T., Dias M., Henry S., Pittman B., Ropchan J., Nabulsi N. (2020). Synaptic Changes in Parkinson Disease Assessed with in vivo Imaging. Ann. Neurol..

[38] Wilson H., Pagano G., De Natale E.R., Mansur A., Caminiti S.P., Polychronis S., Middleton L.T., Price G., Schmidt K.F., Gunn R.N. (2020). Mitochondrial Complex 1, Sigma 1, and Synaptic Vesicle 2A in Early Drug-Naive Parkinson’s Disease. Mov. Disord..

[39] Thomsen M.B., Jacobsen J., Lillethorup T.P., Schacht A.C., Simonsen M., Romero-Ramos M., Brooks D.J., Landau A.M. (2021). In vivo imaging of synaptic SV2A protein density in healthy and striatal-lesioned rats with [11C]UCB-J PET. J. Cereb. Blood Flow Metab..

[40] Thomsen M.B., Ferreira S.A., Schacht A.C., Jacobsen J., Simonsen M., Betzer C., Jensen P.H., Brooks D.J., Landau A.M., Romero-Ramos M. (2020). PET imaging reveals early and progressive dopaminergic deficits after intra-striatal injection of preformed alpha-synuclein fibrils in rats. Neurobiol. Dis..

[41] Groves P.M., Linder J.C., Young S.J. (1994). 5-hydroxydopamine-labeled dopaminergic axons: Three-dimensional reconstructions of axons, synapses and postsynaptic targets in rat neostriatum. Neuroscience.

[42] Uchigashima M., Ohtsuka T., Kobayashi K., Watanabe M. (2016). Dopamine synapse is a neuroligin-2-mediated contact between dopaminergic presynaptic and GABAergic postsynaptic structures. Proc. Natl. Acad. Sci. USA.

[43] Molinet-Dronda F., Gago B., Quiroga-Varela A., Juri C., Collantes M., Delgado M., Prieto E., Ecay M., Iglesias E., Marin C. (2015). Monoaminergic PET imaging and histopathological correlation in unilateral and bilateral 6-hydroxydopamine lesioned rat models of Parkinson’s disease: A longitudinal in-vivo study. Neurobiol. Dis..

[44] Du G., Lewis M.M., Sen S., Wang J., Shaffer M.L., Styner M., Yang Q.X., Huang X. (2012). Imaging nigral pathology and clinical progression in Parkinson’s disease. Mov. Disord..

[45] Kirik D., Rosenblad C., Bjorklund A. (1998). Characterization of behavioral and neurodegenerative changes following partial lesions of the nigrostriatal dopamine system induced by intrastriatal 6-hydroxydopamine in the rat. Exp. Neurol..

[46] Lundblad M., Decressac M., Mattsson B., Bjorklund A. (2012). Impaired neurotransmission caused by overexpression of alpha-synuclein in nigral dopamine neurons. Proc. Natl. Acad. Sci. USA.

[47] Croisier E., Moran L.B., Dexter D.T., Pearce R.K., Graeber M.B. (2005). Microglial inflammation in the parkinsonian substantia nigra: Relationship to alpha-synuclein deposition. J. Neuroinflammation.

[48] Harms A.S., Ferreira S.A., Romero-Ramos M. (2021). Periphery and brain, innate and adaptive immunity in Parkinson’s disease. Acta Neuropathol..

[49] Sanchez-Guajardo V., Febbraro F., Kirik D., Romero-Ramos M. (2010). Microglia acquire distinct activation profiles depending on the degree of alpha-synuclein neuropathology in a rAAV based model of Parkinson’s disease. PLoS ONE.

[50] Theodore S., Cao S., McLean P.J., Standaert D.G. (2008). Targeted overexpression of human alpha-synuclein triggers microglial activation and an adaptive immune response in a mouse model of Parkinson disease. J. Neuropathol. Exp. Neurol..

[51] Barkholt P., Sanchez-Guajardo V., Kirik D., Romero-Ramos M. (2012). Long-term polarization of microglia upon alpha-synuclein overexpression in nonhuman primates. Neuroscience.

[52] Imamura K., Hishikawa N., Sawada M., Nagatsu T., Yoshida M., Hashizume Y. (2003). Distribution of major histocompatibility complex class II-positive microglia and cytokine profile of Parkinson’s disease brains. Acta Neuropathol..

[53] Rodriguez-Chinchilla T., Quiroga-Varela A., Molinet-Dronda F., Belloso-Iguerategui A., Merino-Galan L., Jimenez-Urbieta H., Gago B., Rodriguez-Oroz M.C. (2020). [(18)F]-DPA-714 PET as a specific in vivo marker of early microglial activation in a rat model of progressive dopaminergic degeneration. Eur. J. Nucl. Med. Mol. Imaging.

[54] Crabbe M., Van der Perren A., Kounelis S., Lavreys T., Bormans G., Baekelandt V., Casteels C., Van Laere K. (2019). Temporal changes in neuroinflammation and brain glucose metabolism in a rat model of viral vector-induced alpha-synucleinopathy. Exp. Neurol..

[55] Maia S., Arlicot N., Vierron E., Bodard S., Vergote J., Guilloteau D., Chalon S. (2012). Longitudinal and parallel monitoring of neuroinflammation and neurodegeneration in a 6-hydroxydopamine rat model of Parkinson’s disease. Synapse.

[56] Watson M.B., Richter F., Lee S.K., Gabby L., Wu J., Masliah E., Effros R.B., Chesselet M.F. (2012). Regionally-specific microglial activation in young mice over-expressing human wildtype alpha-synuclein. Exp. Neurol..

[57] Su X., Maguire-Zeiss K.A., Giuliano R., Prifti L., Venkatesh K., Federoff H.J. (2008). Synuclein activates microglia in a model of Parkinson’s disease. Neurobiol. Aging.

[58] Stokholm M.G., Iranzo A., Ostergaard K., Serradell M., Otto M., Svendsen K.B., Garrido A., Vilas D., Borghammer P., Santamaria J. (2017). Assessment of neuroinflammation in patients with idiopathic rapid-eye-movement sleep behaviour disorder: A case-control study. Lancet Neurol..

[59] Iannaccone S., Cerami C., Alessio M., Garibotto V., Panzacchi A., Olivieri S., Gelsomino G., Moresco R.M., Perani D. (2013). In vivo microglia activation in very early dementia with Lewy bodies, comparison with Parkinson’s disease. Parkinsonism Relat. Disord..

[60] Lillethorup T.P., Glud A.N., Landeck N., Alstrup A.K.O., Jakobsen S., Vang K., Doudet D.J., Brooks D.J., Kirik D., Hinz R. (2018). In vivo quantification of glial activation in minipigs overexpressing human alpha-synuclein. Synapse.

[61] Francois C., Percheron G., Yelnik J. (1984). Localization of nigrostriatal, nigrothalamic and nigrotectal neurons in ventricular coordinates in macaques. Neuroscience.

