# Supplementary figures and images for: α-Synuclein Overexpression Increases Dopamine D2/3 Receptor Binding and Immune Activation in a Model of Early Parkinson’s Disease
Source: Biomedicines. 2021 Dec 10;9(12):1876. doi: 10.3390/biomedicines9121876 (PMC8698691; doi:10.3390/biomedicines9121876)

## Supplementary Figure

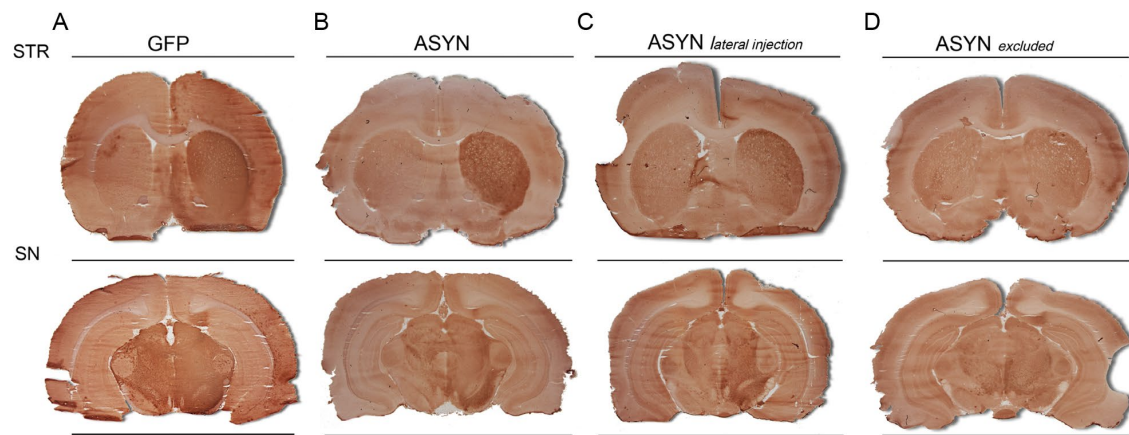

Supplement: Supplementary file 1 [file biomedicines-09-01876-s001.zip › biomedicines-1482204-supplementary.pdf]
